# Electro-mechanical to optical conversion by plasmonic-ferroelectric nanostructures

**DOI:** 10.1515/nanoph-2022-0105

**Published:** 2022-05-30

**Authors:** Artemios Karvounis, Rachel Grange

**Affiliations:** Department of Physics, Optical Nanomaterial Group, Institute for Quantum Electronics, ETH Zurich, Auguste-Piccard-Hof 1, 8093 Zurich, Switzerland

**Keywords:** barium titanate, ferroelectrics, nanocrystals, optomechanics, plasmonics

## Abstract

Barium titanate (BaTiO_3_) is a lead-free ferroelectric crystal used in electro-mechanical transducers and electro-optic films. Nanomechanical devices based on thin films of BaTiO_3_ are still unavailable, as the internal stress of thin ferroelectric films results in brittle fracture. Here, we use the electro-mechanical force to fabricate deformable assemblies (nanobeams) of BaTiO_3_ nanocrystals, on top of plasmonic metasurfaces. The mechanical deformation of the nanobeams is driven by the piezoelectric response of the BaTiO_3_ nanocrystals. The plasmonic-ferroelectric nanostructures due to the plasmonic enhancement enable subwavelength interaction lengths and support reflection modulation up to 2.936 ± 0.008%. Their frequency response is tested across 50 kHz up to 2 MHz and is dependent on the mechanical oscillations of the deformable BaTiO_3_ nanobeams. The ferroelectric nanobeams support mechanical nonlinearities, which offer additional control over the electro-mechanical to optical conversion.

## 1 Introduction

Photonic platforms that couple several degrees of freedom into nanosized systems such as optical, electrical, and mechanical modes are of great interest, as they have become a scheme to study conversion mechanisms, or to deliver active nano-elements for the production of highly responsive optical devices, that can be tuned by external stimuli [1]. The fabrication of nanomechanical resonators up to date has been realised by top–down fabrication methods and led to many breakthroughs in optics and electronics [2, 3]. The most regularly used material for realizing nanomechanical resonators has been silicon and its nitrides, capitalizing the research conducted in CMOS technologies over the past years [4].

However, applications related to optical quantum computing and transduction processes, demand different materials. Despite the effort to transform silicon and other centrosymmetric crystals into noncentrosymmetric through strain engineering and metamaterials [5–7], materials with intrinsic photonic nonlinearities and electro-mechanical response operate even stronger than silicon based devices [8–10]. Ferroelectrics can combine both properties of second-order optical nonlinearities and electro-mechanical responses; therefore render a platform of great interest [11, 12].

Among those, barium titanate, BaTiO_3_ is a lead-free ferroelectric crystal, that combines excellent optical properties such as large optical bandgap (≫3 eV), the highest electro-optic coefficient, (>700 pm/V) considerable high second order susceptibility coefficients 15 pm/V [13] and at the same time it has strong electro-mechanical response (piezoelectric coefficient >300 pC/N) [11]. By electro-mechanical response we refer to the developed mechanical strain, when the crystal is subject to strong polarization fields. Ferroelectrics can be electro-mechanically active both in single crystal or polycrystal form [14], as electrical poling can break the inversion symmetry, whereas the non-ferroelectric ceramics (e.g., ZnO ceramics) show macroscopic symmetry of inversion, cannot exhibit piezoelectricity [14]. Most of the flexible composites of BaTiO_3_ use an organic soft matrix with embedded randomly oriented BaTiO_3_ nanocrystals of different shapes e.g. nanoparticles, nanowires, etc. used for energy harvesting applications, high-k electronic materials, and strain sensors [9, 15, 16]. Recently, single crystal membranes of BaTiO_3_ have been realized with exceptional elasticity, where domain reformation could compensate the mechanical stress and avoid fracture [17]. However, there are no reports of nanodevices that harness the nanomechanical motion to serve as tunable optical components, despite the recent progress done in fabrication.

The research over thin films with tunable optical properties is an active field in nanophotonics, and it is realised with various mechanism and material platforms related to liquid crystals [18, 19], heavily doped semiconductors [20], phase change materials [21], nanomechanical systems [22], chromic effects [23, 24], and electro-optic films [25]. Here, we show that electro-mechanical strain developed on a thin film of randomly oriented BaTiO_3_ nanocrystals upon intense electric fields, can lead to the formation of flexible, nanomechanical resonators. These flexible resonators consist of BaTiO_3_ nanocrystals and form nanobeams adjacent to a plasmonic metasurface. Their nano-motion can modify the optical properties of the plasmonic-ferroelectric nanostructure. We characterise the frequency response of the proposed device across the first three mechanical eigen frequencies and record mechanical nonlinearities associated with the electro-mechanical effect.

## 2 Results & discussions

### 2.1 Fabrication

**Figure 1:**
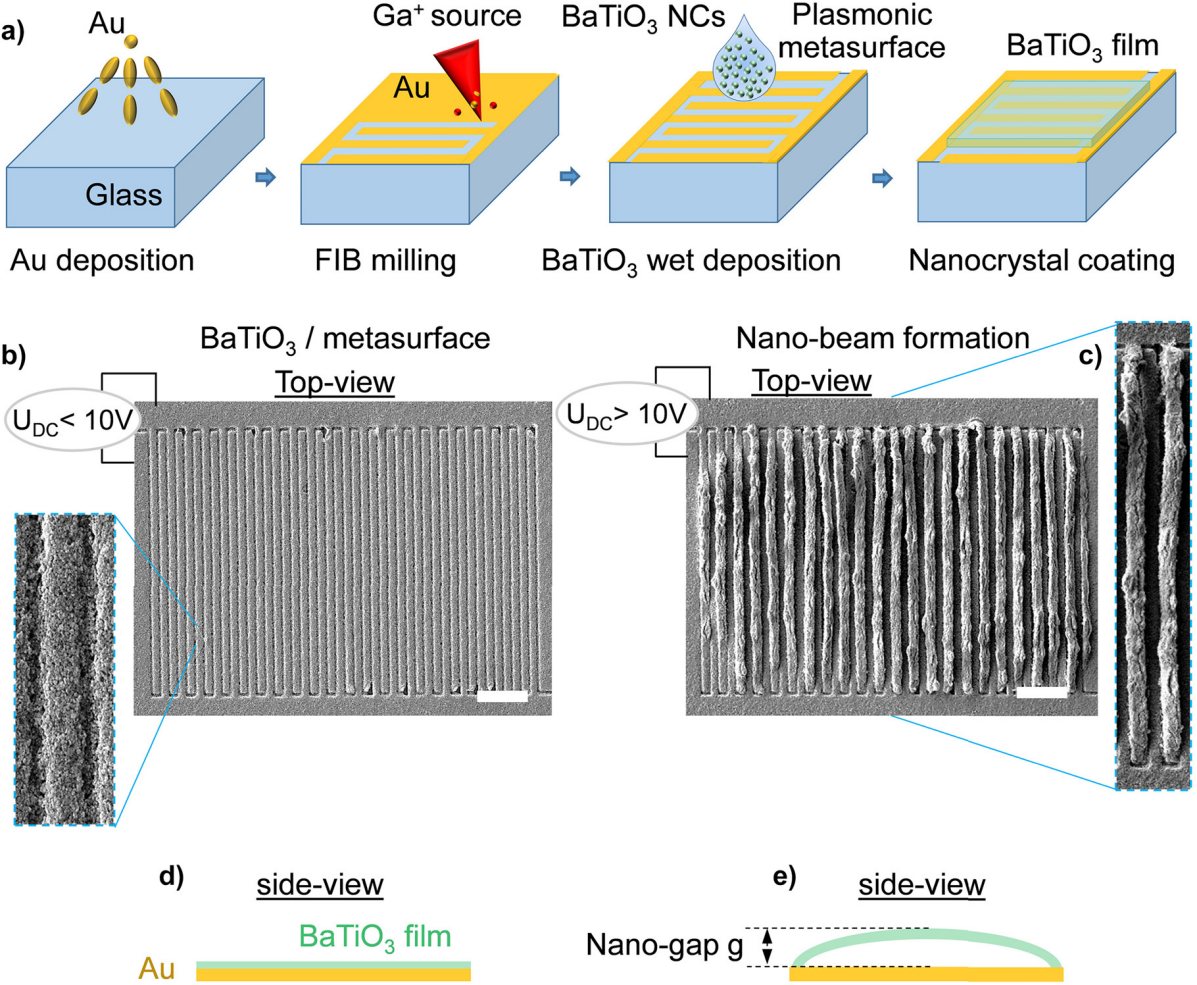
Fabrication steps of the deformable assemblies of BaTiO_3_ nanobeams. (a) Fabrication procedure of the randomly oriented thin film of BaTiO_3_ nanocrystals on top of gold metasurface. A gold film of 200 nm thickness is deposited on glass by e-beam evaporation and subsequently nanostructured by focused ion beam (FIB) milling. Next, we use suspended BaTiO_3_ nanocrystals (average diameter 50 nm) in water solution and deposit them by drop-cast/spin-coating, producing a BaTiO_3_ film on top of the plasmonic metasurface. (b) Top-down SEM image of the plasmonic-ferroelectric nanostructure; samples remain intact for bias voltage lower than 10 V, scale bar 3 μm. The SEM inset shows a section of the sample under higher magnification. (c) Top-down SEM image of the plasmonic-ferroelectric nanostructure after the application of 10 V; SEM shows the mechanical buckling of the BaTiO_3_ nanocrystals actuated from the high potential gold nanowires, scale bar 3 μm. The SEM inset shows a pair of buckled nanobeams. (d and e) Side view schematic before and after the nanobeam formation, with the corresponding nanogap annotated as *g*.

The fabrication process is described in Figure 1a. We deposit 200 nm thick gold (Au) films on optically flat glass substrates by e-beam evaporation. Next, we nanostructure the gold films by focus ion beam milling (FIB) to fabricate a periodic array of gold nanowires that form the plasmonic metasurface. Each pair of gold nanowires is connected alternately to two electrical terminals, on opposite sides of the device. After ozone treatment of the samples for 10 min, we drop cast aqueous solution with 25% wt concentration of BaTiO_3_ nanocrystals (average diameter 50 nm) and spin-coat with 5000 rpm to create a flat coating of randomly oriented BaTiO_3_ nanocrystals (coating thickness: 100 nm) on top of the plasmonic metasurface. Electrical DC signals (*U*_DC_) up to 10 V do not form the flexible nanobeams, however above this voltage the polarisation fields lead to the mechanical buckling of the BaTiO_3_ nanocrystals located on top of the high potential gold nanowires. Thus, it formed an array of flexible nanobeams with the double periodicity of the plasmonic metasurface. Electro-mechanically assembled BaTiO_3_ nanobeams can reach length up to 18 μm. Bias voltage higher than 15 V result in the electrical breakdown of the samples. All the fabrication steps and the corresponding scanning electron microscope images before and after the formation of the BaTiO_3_ nanobeams are shown in Figure 1.

Plasmonic nanostructures have been used in several reports to convert the nanomechanical motion into optical modulation actuated either by electrical or thermal methods [26–28]. Here, we use a plasmonic metasurface made of a subwavelength periodic array of gold nanowires. All gold nanowires have a width of 380 nm and the period of the plasmonic metasurface is 500 nm. Each pair of nanowires is connected alternately to two electrical terminals on opposite sides of the device and excites the nanomotion of the BaTiO_3_ nanobeams. The device geometry is represented by a scanning electron microscopy (SEM) image in Figure 1b with total dimensions of 18 μm × 24 μm. In such configuration, the periodic array of the gold nanowires supports plasmonic resonances within the vicinity of the BaTiO_3_ nanobeams.

### 2.2 Electro-mechanical to optical conversion

**Figure 2:**
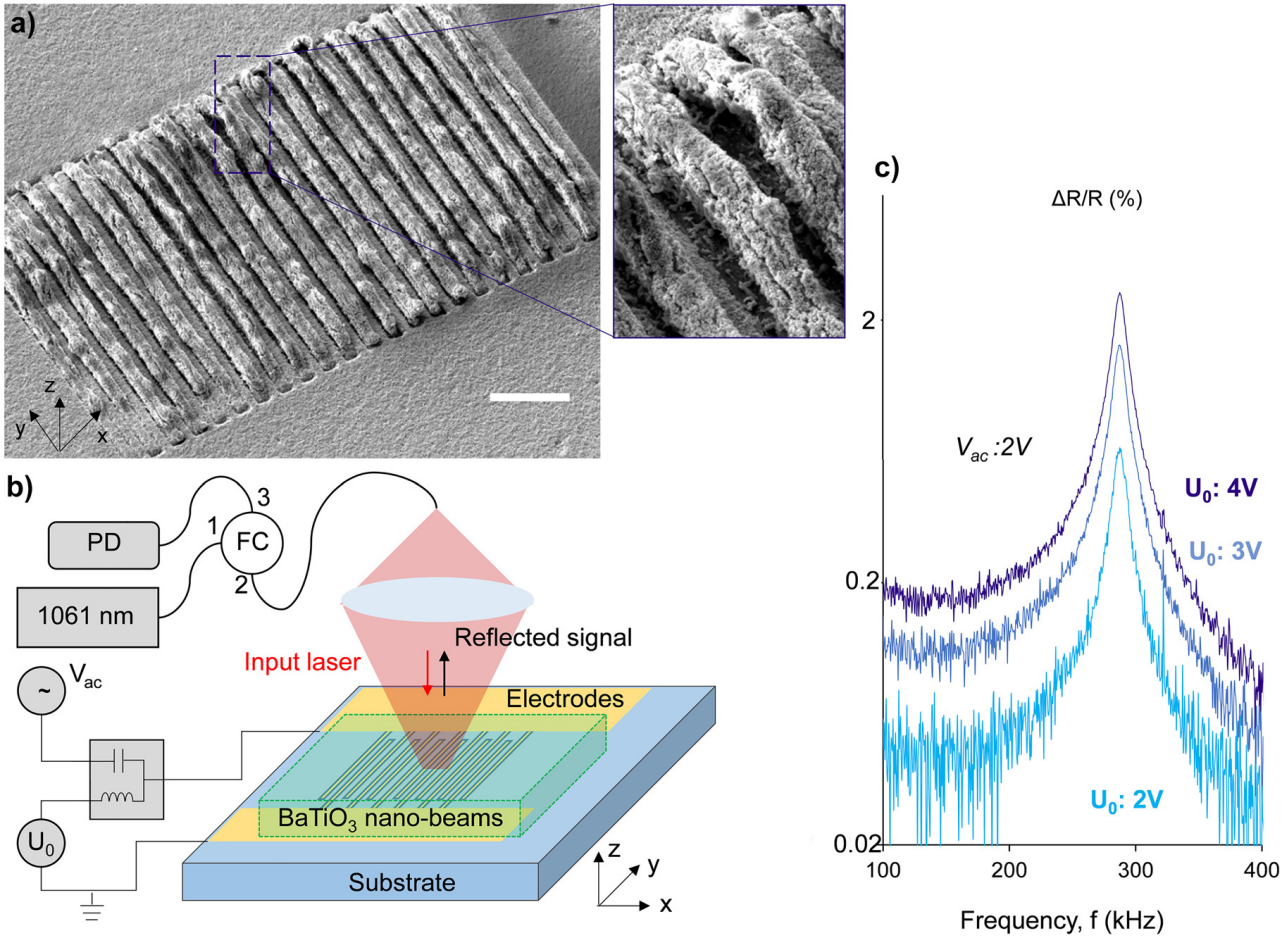
Optical modulation of the plasmonic-ferroelectric nanostructure actuated by piezoelectric force. (a) Oblique SEM image of the BaTiO_3_ nanobeam array adjacent to the plasmonic metasurface. Scale bar 3 μm. Inset: close view of the deformable BaTiO_3_ nanobeams detached from the plasmonic metasurface. (b) Schematic of the measurement setup for the actuation of the electro-mechanical BaTiO_3_ nanobeams. The bias tee allows the simultaneous application of DC and AC signals to the samples. Input wavelength of the laser beam is at 1061 nm and polarised along the *x*-axis. All measurements performed at room temperature and at ambient pressure. PD: photodetector, FC: fiber circulator. (c) Dependence of reflected optical signal upon driving frequency, across the first mechanical resonance mode. The AC signal is constant at 2 V and each line corresponds to different DC bias level, as annotated. Amplitude of the optical signal is plotted in logarithmic scale.

We test the electro-mechanical to optical conversion for the sample shown in the Figures 1c and 2a. The nanogap *g* is formed between the gold nanowires and every second deformable BaTiO_3_ nanobeam, see Figure 1c and e. Samples that have not been subject to the electro-mechanical actuation (e.g. *U*_o_ 10 V), do not show optical modulation of the reflected signal. A voltage *U*_DC_ larger than 10 V is used to initialize the device by releasing the ferroelectric nanowires and then, once released, only a few volts (*U*_o_) suffice to perform modulation. A schematic of the experimental setup is presented in Figure 2b. The input laser beam (central wavelength 1061 nm) on the sample is launched by a low-noise, fiber-coupled diode laser. We keep the optical power steady and relatively low, below 0.5 mW, as we want to avoid thermal related effects. A pair of lenses is used to collimate and focus the laser beam on the sample, with a beam spot size of 2–3 μm. The reflected signal is then collected from a fiber circulator and it is directed in an InGaAs amplified photodiode. We bias electrically the sample with a DC offset, *U*_0_ produced by a power supply and an AC signal, *V*_ac_. The resistance of the sample is measured to be 100 MΩ, therefore a bias tee is employed to deliver efficiently both signals on the sample. The *V*_ac_ is produced by a lock-in amplifier and an RF amplifier; it has a sinusoidal waveform and reaches the value of 2 V peak-to-peak. The photodiode is also connected to the input port of the lock-in amplifier. The frequency scan along the first mechanical eigenfrequency is presented in Figure 2c. The amplitude of the recorded signal is dependent on the DC offset applied to the sample and it is increasing as we increase the *U*_0_. We record the reflection modulation up to 2.385 ± 0.008%.

### 2.3 Optical characterization & simulation

**Figure 3:**
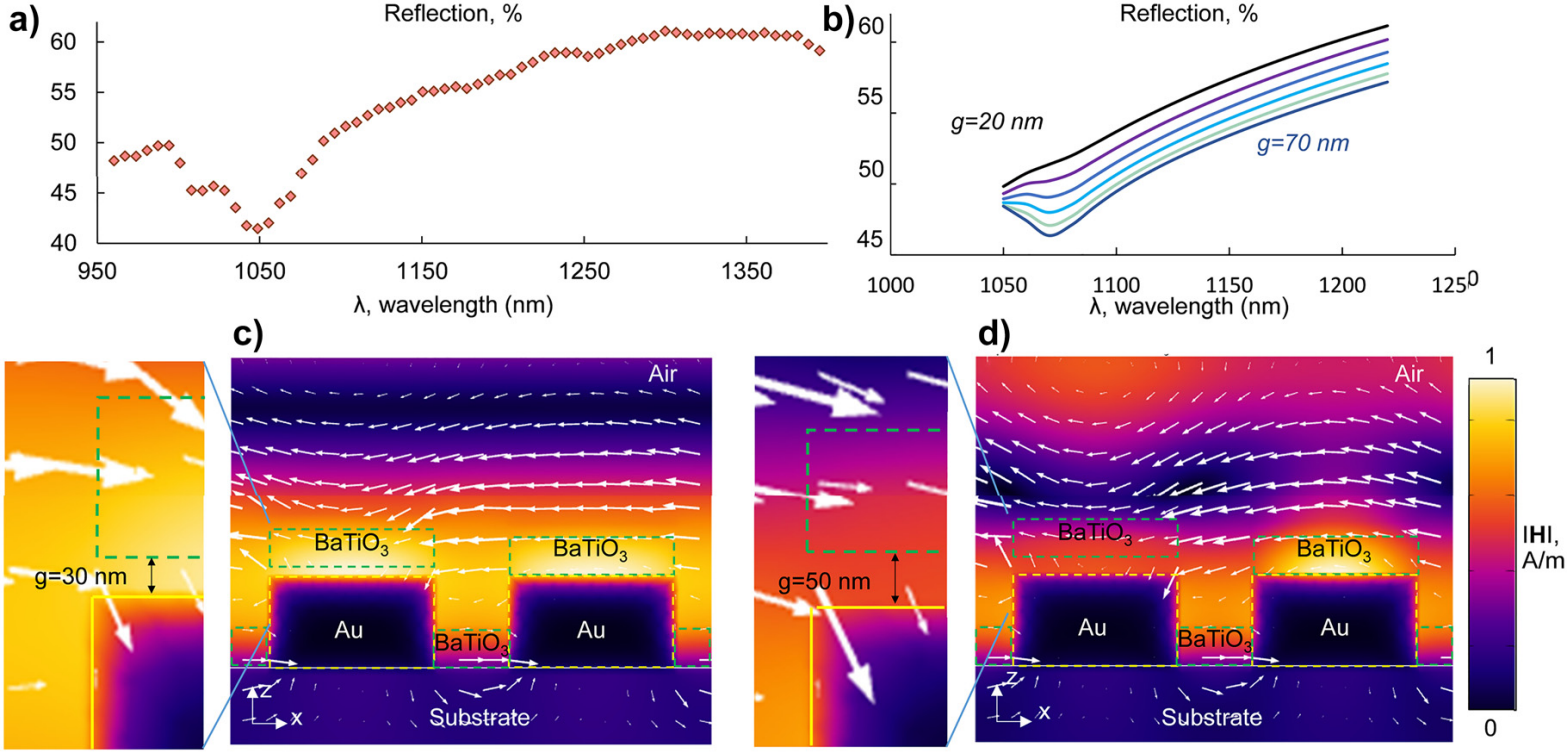
Impact of the nanomechanical motion on the optical properties of the ferroelectric-plasmonic nanostructure. (a and b) Experimental and simulated reflection spectra of the plasmonic-ferroelectric nanostructure. (b) Simulated spectra for various nanogap size (*g*) from 20 nm up to 70 nm in steps of 10 nm, as annotated. (c and d) The |H|-field distribution in the *x*-*z* plane, overlaid with arrows denoting the direction of the electric field, for one pair of gold nanowires and BaTiO_3_ nanobeams. (c) The deformable BaTiO_3_ nanobeam forms a 30 nm nanogap (*g*) with the gold nanowire, in (d) the nanogap is increased to 50 nm. In both color maps, input light is considered as plane wave with the polarisation vector along *x*-direction.

Similar nanomechanical systems are based on displacements in the range of pm up to nm, where optical cavities or resonant structures are used to enhance the detection of the nanomotion from flexible parts [26–28]. Here, the plasmonic metasurface offers the increased sensitivity needed. At Figure 3a, we present the experimental reflection spectra of the plasmonic-ferroelectric nanostructure obtained using an infrared camera connected to Andor spectrograph. A sampling aperture of 10 × 10 μm^2^ is used while a 50× objective was used to focus and collect the reflected signal from a linearly polarized halogen lamp. Data were normalized to reference levels of a silver mirror (high reflector) and averaged over 15 repeated measurement cycles, each with a 200 ms integration time. We plot the experimental reflection of the nanostructure in Figure 3a and the numerically calculated with finite element methods, reflection over various nanogap sizes *g* in Figure 3b. We numerically simulate a pair of gold nanowires covered by a BaTiO_3_ layer of 100 nm and a deformable BaTiO_3_ nanobeam. For this simulation we use the same refractive index for the BaTiO_3_ nanocrystals and gold as in [29]. The width of gold nanowires and BaTiO_3_ nanobeams are 350 nm and a groove of 150 nm wide separates them. In our model, the excitation port is a linearly polarised plane wave, propagating along the *z*-axis with the polarisation vector lying along the *x*-axis, at the wavelength of 1061 nm. We present the color maps for a cross-section of two different nanobeam displacement (Figure 3c and d), where the deformable BaTiO_3_ nanobeams form a nanogap *g* above the gold nanowires, equal with 30 nm and 50 nm, respectively. The |H|- field distribution at the wavelength of 1061 nm indicates that light is localized on the top surface of both gold nanowires for 30 nm displacement, while for larger displacements, namely 50 nm |H|- field distribution is reduced below the deformable BaTiO_3_ nanobeam. In our simulation, we consider a uniform displacement of periodic (over *x*-axis) and infinitely long nanobeams (over *y*-axis). The reflection spectra are reduced by almost 5% for every 10 nm of displacement, see Figure 3b. However, the actual displacement of the nanobeams is not uniform, as in the central part of the samples there is more space for the nanobeams to move than in the edges of the sample, where they are fixed, as well as the nanobeams are not perfectly periodic. This explains why we record smaller reflection changes, than those we simulate.

### 2.4 Frequency dependent response

**Figure 4:**
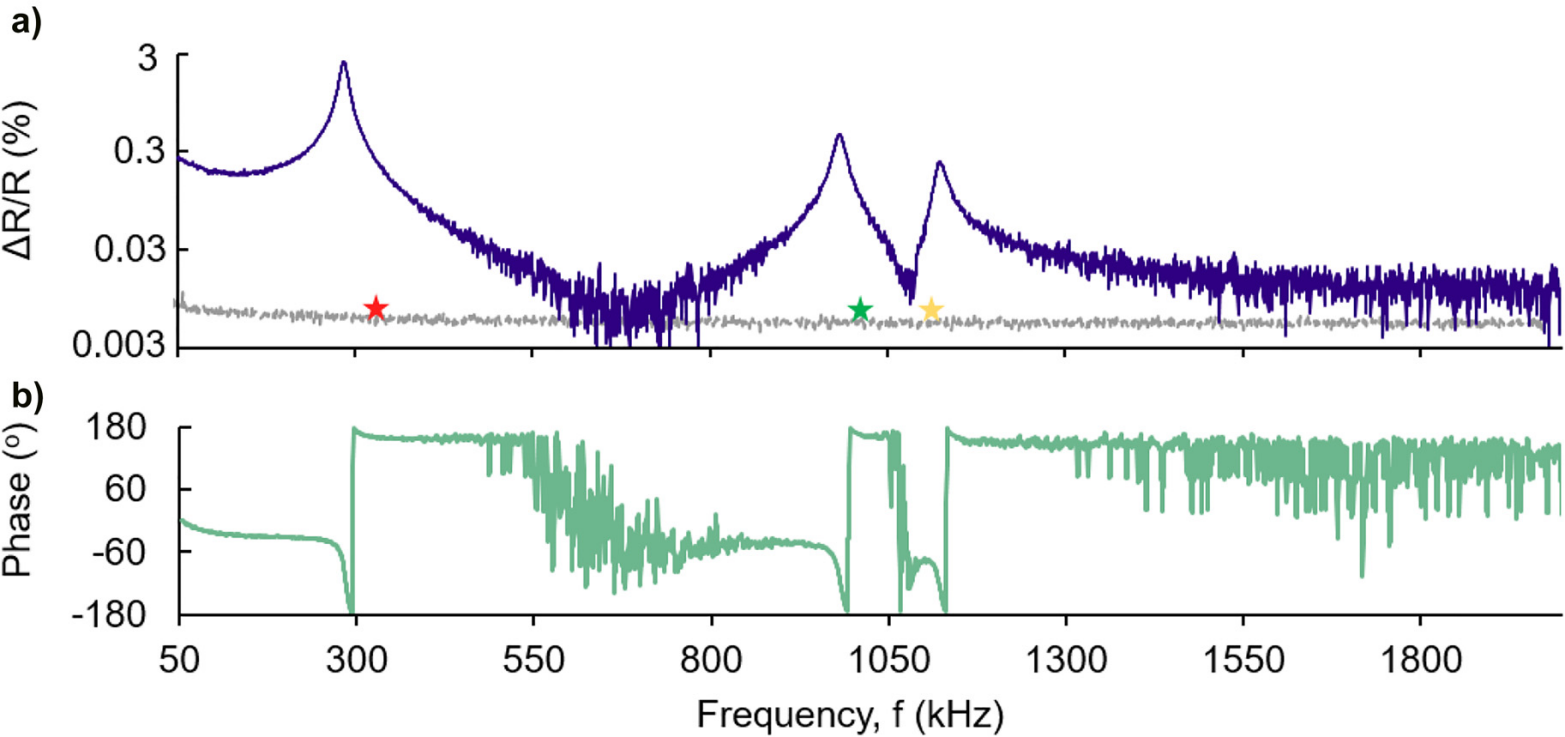
Reflection modulation of the defromable BaTiO_3_ nanobeams as a function of the driving frequency. (a) Reflection spectrum of the plasmonic-ferroelectric nanostructure, blue line. Three mechanical resonances are recorded at 285 kHz, 983 kHz, and 1.13 MHz. Numerically calculated values are annotated with red, green and yellow star, respectively. Samples are biased at *U*_o_: 7 V and *V*_AC_: 2 V peak to peak. Reflection axis is in logarithmic scale. Grey line: the signal recorded for a reference sample without any BaTiO_3_ nanocrystal. (b) Corresponding phase spectra measured between the input (electric) and detected (optical) signal.

The experimental response of the samples while varying the frequency *f* of an applied sinusoidal electrical bias from 50 kHz up to 2 MHz is presented on Figure 4. The samples are first subject to a DC offset of 10–12 V prior to measurement, where several BaTiO_3_ nanobeams have detached from the plasmonic metasurface. The sample is biased with a fixed DC offset of 7 V and fixed AC signal peak-to-peak of 2 V. All experiments are performed in an open environment, in ambient temperature and pressure. At the low frequencies, the induced displacement of the nanobeams is relatively constant and therefore the magnitude of the induced change in reflection is measurable. As we increase the driving frequencies at the mechanical eigenmodes the reflection signal is enhanced. Experimentally these values correspond at the frequencies of 293 kHz, 0.98 MHz, and 1.13 MHz, respectively. The electro-mechanical response of the proposed device is much broader to other nanomechanical systems, as the Q-factors of the mechanical motion of the BaTiO_3_ nanobeams are in the order of 20–30, *Q* = *f*_res_/Δ*f*. We argue that this response is due to two parameters. Firstly, in our system we have more than one resonators vibrating, as within the laser beam can fit 2–3 BaTiO_3_ nanobeams that can modulate the reflected signal, therefore induce homogeneous broadening of the mechanical resonance. Secondly, the measurement is conducted under ambient pressure, therefore the damping from air leads to larger modulation bandwidth.

The mechanical and geometrical properties of the BaTiO_3_ nanobeams define the eigenfrequencies that maximise the modulated signal. The Young modulus of BaTiO_3_ polycrystalline films is reduced in comparison to the values of the bulk single crystal, namely 60 GPa [30]. Here, we model the mechanical behavior of the nanobeams by assuming a Young modulus of 3 GPa as reported in [9]. The three mechanical eigenfrequencies are estimated based on finite element method calculations to be 360 kHz, 1.02 MHz, and 1.07 MHz. The deviation between the experimental and numerically calculated values is due to our limitation to define the exact Young’s modulus of the randomly oriented ferroelectric nanocrystals. However, they give a qualitative description of the nanobeam motion.

### 2.5 Mechanical nonlinearity

**Figure 5:**
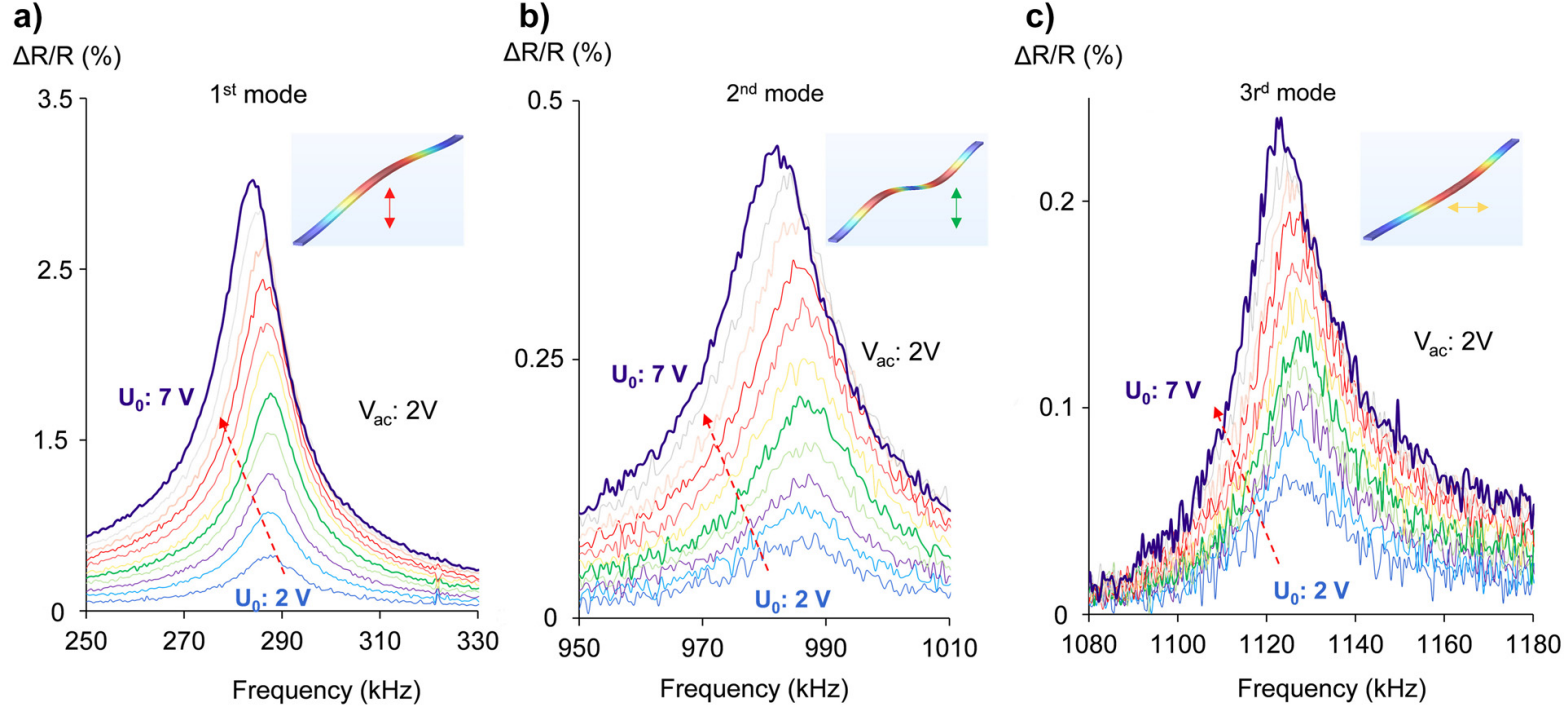
Mechanical nonlinearities in photonic electro-mechanical assemblies of BaTiO_3_ nanocrystals. Signal recorded for gradual increment of DC offset, *U*_0_ in steps of 0.5 V, as indicated by the red arrow. (a) First mechanical eigenmode corresponds to the out-of-plane displacement of the BaTiO_3_ nanobeams. (b) Second mechanical eigenmode corresponds to the out-of-plane displacement of the BaTiO_3_ nanobeams, (c) third mechanical eigenmode corresponds to the in-plane displacement of the BaTiO_3_ nanobeams. Insets in (a), (b), and (c) show the FEM simulations of the associated mechanical modes. Red colored parts of the nanobeams represent maximum deformation, and blue no deformation. Arrows denote the direction of the nanobeam motion.

Next, we present the mechanical nonlinearities associated with the vibration of the BaTiO_3_ nanobeams subject to a gradually increasing DC offset signal *U*_0_. The modulation amplitude is recorded in steps of 0.5 V, while the AC signal *V*_ac_ is kept constant at 2 V. The experimental spectra are presented in Figure 5. In the Figure 5a, we present the first mechanical eigenmode that corresponds to the out-of-plane displacement. This mode has the larger impact over the optical properties of the plasmonic-ferroelectric nanostructure, reaching the value of 2.936 ± 0.008%. The mechanical eigenfrequencies of the vibration modes are not dependent up to a DC offset of *U*_0_: 4 V, for larger values the resonant frequency is contracted by almost 1 kHz/V, reduced from 288 kHz down to 285 kHz. This response is explained by the reduction of the Young modulus, as the mechanical load over the BaTiO_3_ nanobeams is increased, the effect called mechanical softening of the nanobeams is taking place [4]. Similarly this is the mechanical behavior recorded for the higher modes as well. For the second and third mode, the signal is noisier as the impact of these modes on the total optical properties in the proposed devices is reduced. The second mode is shifted from 986 kHz to 983 kHz, similar to the first mode. The third mode is reduced from 1127 kHz down to 1122 kHz, that corresponds to a shift of 1.7 kHz/V. We argue that the difference between the rates of the resonance shift can be explained as the different direction of displacement of the nanobeams, as the FEM mechanical calculation show the third mode corresponds to the in-plane vibration mode. The electrical control of the resonant frequencies of each mode, render an extra parameter for the active tuning of these devices.

## 3 Conclusions

In conclusion, we have presented a novel platform to produce electro-mechanical assemblies of BaTiO_3_ nanocrystals with application in the conversion of the electro-mechanical motion into resonant modulation of optical properties. The actuation voltages are lower than other electro-mechanical materials. As an example, electronic polymers such as electrostrictive, dielectric elastomers, piezoelectric, and ferroelectric polymers require high activation fields (over than 150 V μm^−1^) [31], while the plasmonic-ferroelectric nanostructures require activation fields of 10 V μm^−1^. The proposed mechanism is very strong, given the short interaction length of these photonic devices, namely less than 5 times the operational wavelength. We tested the operation over several minutes without any breakdown of the samples, if actuation voltages remain within specified limits. Therefore, we anticipate that the plasmonic-ferroelectric can be an enabling platform for electrically actuated optical modulators, as they outperform in terms of size over linear electro-optic modulators. The strong electro-mechanical coefficient of BaTiO_3_ outperforms several materials such as silicon, quartz, LiNbO_3_, or GaAs, therefore it can be used in microwave-to-optical transduction schemes [32]. Furthermore the second order nonlinearity of BaTiO_3_ can be utilized to produce entangled photon pairs via spontaneous parametric down-conversion. As a result, we anticipate the research to shift towards ferroelectrics for quantum applications. Moreover, the samples do not show any thermal instability, as there are no running currents through the nanodevices and the power of the laser beam was kept below 0.5 mW. We propose that different design with shorter moving parts could lead to higher modulation frequencies. Device breakdown happens for electrical signals stronger than 15 V. In terms of speed, shorter moving parts, could improve the modulation speed of the devices. Apart from tunable photonic components for free space applications, such devices could be used in application related to laser physics, such as Q-switching or in the field of sensing, such as strain sensors.
